# Kernel density weighted loess normalization improves the performance of detection within asymmetrical data

**DOI:** 10.1186/1471-2105-12-222

**Published:** 2011-06-01

**Authors:** Wen-Ping Hsieh, Tzu-Ming Chu, Yu-Min Lin, Russell D Wolfinger

**Affiliations:** 1 Institute of Statistics, National Tsing Hua University, Hsin-Chu City, 300, Taiwan; 2 SAS Institute Inc., Cary NC 27513, USA

## Abstract

**Background:**

Normalization of gene expression data has been studied for many years and various strategies have been formulated to deal with various types of data. Most normalization algorithms rely on the assumption that the number of up-regulated genes and the number of down-regulated genes are roughly the same. However, the well-known Golden Spike experiment presents a unique situation in which differentially regulated genes are biased toward one direction, thereby challenging the conclusions of previous bench mark studies.

**Results:**

This study proposes two novel approaches, KDL and KDQ, based on kernel density estimation to improve upon the basic idea of invariant set selection. The key concept is to provide various importance scores to data points on the MA plot according to their proximity to the cluster of the null genes under the assumption that null genes are more densely distributed than those that are differentially regulated. The comparison is demonstrated in the Golden Spike experiment as well as with simulation data using the ROC curves and compression rates. KDL and KDQ in combination with GCRMA provided the best performance among all approaches.

**Conclusions:**

This study determined that methods based on invariant sets are better able to resolve the problem of asymmetry. Normalization, either before or after expression summary for probesets, improves performance to a similar degree.

## 1. Background

The normalization of data is a crucial step in the analysis of microarray data. The main purpose is the removal of systematic variations, while preserving biological variations of interest. Most of the algorithms that have been proposed and utilized over the years are based on reasonable assumptions that are generally true for real data and large scale studies.

Quantile normalization [1] and loess normalization [2] are among the most popular approaches, and included as built-in, standard procedures in most software packages. Motivated by the idea of a Q-Q plot, quantile normalization makes the distribution of probe intensities identical for each array. The assumption of equal distribution is so strong that it is often criticized for causing violations in a number of applications. Nevertheless, quantile normalization remains one of the most widely used methods, due to its computational efficiency and low degree of variation across samples. Loess normalization generalizes the M vs. A method presented by Dudoit et al., by performing local regression for each pair of arrays [3]. M and A represent the difference and average of the log transformed intensities in each pair of arrays, respectively. Although the M vs. A method was originally proposed for two-color arrays, it has also been applied to single color arrays, with considerable success. However, the performance of loess normalization relies heavily on the assumption that the gene effect is symmetrical with respect to increases or decreases in expression levels.

The aforementioned assumptions could be seriously biased under certain conditions. One well accepted condition is that a moderately sized group of genes is enhanced or suppressed in the same direction, invalidating the common assumption of equal up- and down-regulation [4,5], as occurs in cross-species hybridization [6,7]. When arrays are applied to strains that are inconsistent with the strain used to design the array, the probe intensities are all relatively lower than the standard strain at polymorphic sites. Regardless of whether this is due to true differences in expression or hybridization strength, genome-wide expression distribution will be biased if there are a moderate number of polymorphic sites. SNPscanner [8] considers this issue in the normalization step; however, it simply selects probes that do not deviate a great deal from the median, the criterion for 1.5 standard deviation. The other situation requiring attention is small arrays tailored to specific applications [9]. This violates the common assumption that most genes are not differentially expressed across samples. Typically, all of the genes in small scale arrays are crucial to specific purposes; hence, they are likely to change at different scales and might change in the same direction.

Another group of normalization methods is based on invariant sets, which are selected as probes that are not differentially expressed across conditions. An invariant set is used to form the standard curve for intensity based normalization [5,10]. Invariant sets are less sensitive to the problem of non-symmetrical distribution of gene effects. An invariant set can be defined biologically as housekeeping genes or computationally as genes with roughly the same ranking across arrays [5,10]. The former strategy selects genes that are known to be expressed at a constant level in various tissues and conditions. Unfortunately, extensive knowledge is required to define the genes in this category and well-established genes are seldom adequate to cover the entire expression range of the array. A number of studies have reported failures using this strategy [11-14], because there is no guarantee that a well known housekeeping gene will maintain its expression pattern under the conditions encountered in novel research.

The computational derivation of invariant sets can be traced back to the work of Li and Wong [10]. This approach relies on the ranking of genes within each array. If a gene is ranked equally in each of the arrays, it is a perfect candidate for an invariant set. However, when a significant proportion of the genes are differentially expressed and when the effects are biased in the same direction, there could be a global shift in ranking [5]. Hence, there remains an implicit assumption of symmetry. Pelz et al. [5] approached this issue through an iterative process, by recursively removing the gene with the highest variation in ranking, and re-ranking the genes in each array, following each removal. This approach is claimed to be less sensitive to asymmetry in gene effects.

Ni et al. moved away from rank-based methods, identfying an invariant set using a two-step kernel method that is not sensitive to asymmetry [15]. However, the success of the iterative two-step approach depends heavily on the choice of initial seed, because the invariant sets are derived sequentially throughout the range of expression. Moreover, their algorithm requires several individual steps and heuristic settings.

In general, normalization methods based on invariant sets are performed in three-steps: selection, curve fitting, and scoring. Usually selection is based on heuristic criteria determining the cutoff for inclusion or exclusion of probes in an invariant set. This step is essential if this method is to outperform regular global normalization methods. In the second step, normalization curves are fit, such that the intensity-based patterns can be described using only the probes included in the selected invariant set. In the third scoring step, the fitting results from the second step are extended to the probes not included in the selected invariant set. The range of probe intensity in the selected invariant set does not necessarily cover the entire range of intensities among all of the probes. Scoring the data lying outside the range of the invariant set is a statistical issue.

This study proposes a Kernel Density weighted Loess (KDL) method, adopting a concept similar to the detection of invariant sets through the estimation of density. KDL methods are carefully configured for single color arrays, particularly on the Affymetrix platform. However, there are no specific settings for the format of arrays; therefore, it is well suited to fitting the data of two-color arrays. Although Ni et al. estimated density based only on M signals, our approach employs two-dimensional kernel density estimation for both the M and A, simultaneously. This eliminates the need to bin the data or introduce an iterative procedure. Our proposed approach was first implemented as the soft criterion described in Section 5.2 to improve general loess normalization. The estimation of density provides different weights for each data point in the subsequent loess normalization. The estimation of density can also be used to select invariant genes, as described in Section 5.3, thereby integrating the idea of invariant sets with quantile normalization.

Normalization should generate data that reflects true variations in gene expression, to ensure that the subsequent step of detecting differentially expressed genes functions correctly and returns a good estimate of fold change. In Section 2.2, we compare the detection power of popular normalization methods such as loess, quantile, and invariant set based methods, including dChip, GRSN, and the proposed kernel density weighted loess (KDL) and kernel density based quantile (KDQ) normalization methods. Performance was assessed using ROC curves applied to the Golden Spike experiment and simulation data. The compression rate between the expected fold change and the observed fold change is investigated in Section 2.3. The proposed methods provide the largest area under the ROC curves and the lowest compression rate.

## 2. Results

### 2.1 Asymmetric gene effects and empty set issues

**Figure 1:**
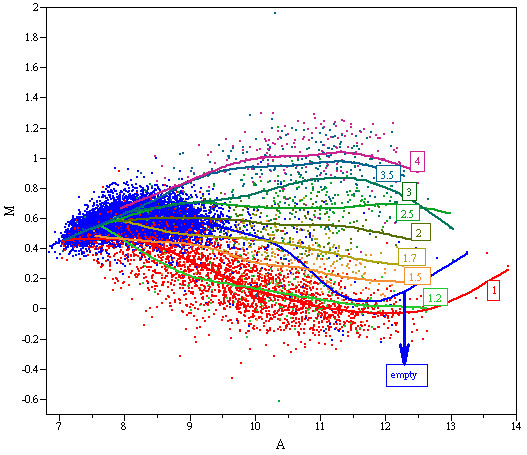
**MA plot for S and C samples Each probe set is first summarized with the median intensity**. The three constant samples are averaged as the C signals while the three spike-in samples are averaged as the S signals. The MA plot is then plotted with the difference between S and C versus the average of S and C. The color scheme is associated with the nominal fold change. Genes of the same fold change are fitted with a smoothing spline curve.

The Golden Spike experiment [4] consists of six arrays separated into spike (S) and constant (C) groups with three replicates each. A total of 3866 RNA transcripts of known concentration were present in the solution. Among these transcripts, 2,535 were assigned equal concentration in S and C conditions, while the concentration of the other 1,331 were increased in S relative to C. Consequently, 10,144 probe sets in the array were expected to be "empty" with no signals. The MA plot between the average intensity levels for both conditions is shown in Figure 1.

The MA plot in Figure 1 illustrates at least two issues that have been widely discussed with regard to Golden Spike data. The first issue concerns empty genes, which have been observed to behave differently from spiked-in genes with one-fold change [16]. Most of the empty genes have higher M values than spiked-in genes with one-fold change. This is the major reason for the non-uniform distribution of null genes [17-19]. Prevailing empty genes have never been observed in real applications, because it is not known *a priori *whether a gene has shut down completely. However, the existence of "empty genes" might be the result of either non-functioning genes or bad probes. As far as we know, there is no specific methodology for automatically removing empty probes. A number of studies have suggested removing known empty sets when comparing methodologies using Golden Spike data [15,20], and we followed this strategy to ensure a fair comparison. All comparisons of the full dataset are presented in the additional documents.

**Figure 2:**
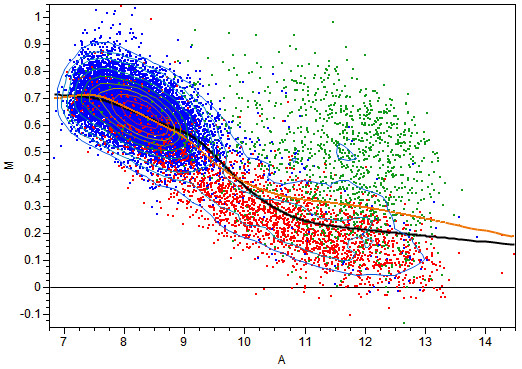
**MA plot between the reference and the third replicate of S group The blue spots are the empty genes and the red ones are the 1× genes**. The green genes have higher concentration in S group than in C group. The black curve is fitted with KDL while the orange curve is fitted with regular loess.

The heterogeneity between the two groups of null genes appears to be well resolved in Figure 2, in which the MA plot for one of the arrays in the S group is displayed. Obviously, the M values are above the zero horizontal line, and this is an intrinsic characteristic of the design. The kernel density contour is also plotted and all of the null genes fall into the high density region. The KDL curve captures the center of the null genes smoothly; therefore, the adjustment is expected to be more reasonable than the global adjustment using regular loess.

**Figure 3:**
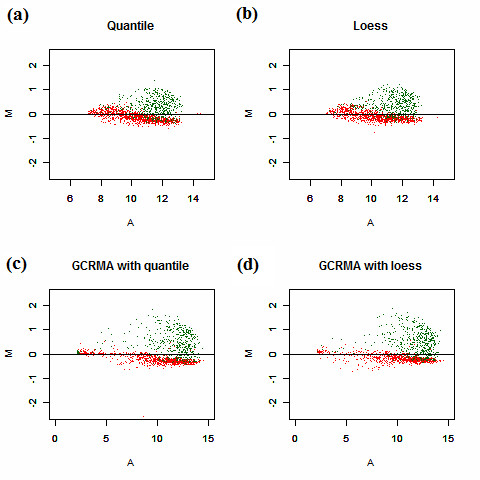
**MA plots for probe-level normalization using data without the empty genes**. (a) Data are normalized with quantile normalization and summarized with the median of each probe set. (b) Data are normalized with loess normalization and summarized with the median of each probe set. (c) Data are first background-corrected with GCRMA and normalized with quantile normalization. The expression summary is then computed using median polish. (d) Data are first background-corrected with GCRMA and normalized with loess normalization. The expression summary is then computed using median polish. The red points are the 1× genes and the green ones are spiked with higher concentration in the S group than in the C group.

The second issue in Figure 1 deals with increased signals for spiked-in genes in the S group. This violates the assumptions of most traditional normalization methods, often leading to failure when using those methods [15-17,21]. As shown in Figure 3 and Additional file 1, when the data are normalized with loess or quantile normalization, the null genes cannot be adjusted to the horizontal line at zero. The difference between S and C for most of the genes is underestimated, particularly at the high end. Previous studies have shown that general normalization methods are capable of correcting for noise, but they do not work well for global asymmetric patterns, as in this experiment. No systematic assessment is available to determine how frequently asymmetry occurs in practice or the extent to which it is present. Nevertheless, a number of applications are intrinsically endowed with such a characteristic and should be handled carefully. These applications include cross-species hybridization and expression arrays for small sets of specific targets.

**Figure 4:**
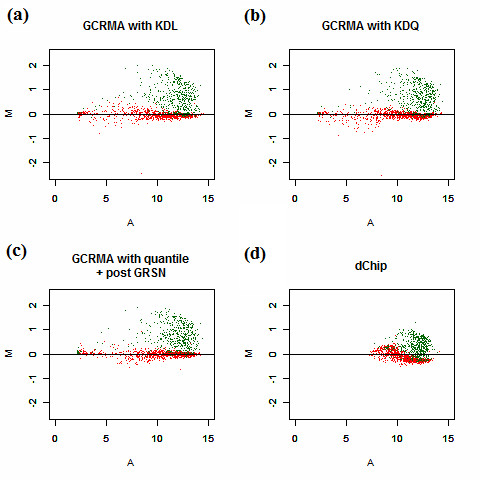
**MA plots for invariant set based normalization methods using data without the empty genes**. (a) Data are background-corrected with GCRMA and normalized at the probe level with KDL. Median polish is used to summarize the probe set expression level. (b) The same as (a) while replacing the normalization with KDQ. (c) Data are background-corrected with GCRMA and normalized at the probe level with quantile. Median polish is used to summarize the probe set expression level. The data are then normalized again at the post summary level with GRSN. (d) Li and Wong's dChip method implemented in R.

Figure 4 and Additional file 2 present a comparison of results from the methods based on invariant sets, including probe-level normalization with dChip, KDL, KDQ, and post-summary normalization with GRSN. The Model Based Expression Index (MBEI) derived from dChip is log2 transformed to parallel the other methods. Most of these methods, with the exception of dChip, get 1× genes well aligned with the zero horizontal line when removing empty genes. The large effect of S relative to C and the asymmetry towards positive differences appears to alter the global ranking of genes across all of the arrays, resulting in bias in dChip. Large variations at low intensity values make dChip a less favorable choice for the detection of differentially expressed genes in this scenario (Figure 4(d) and Additional file 2, Figure S2(d)). GCRMA is well known for its compression of noise at low intensity levels, employing quantile normalization at the probe level. When this normalization is replaced by KDL normalization, the 1× genes lie nearly on the zero line (Figure 4(a) and Additional file 2, Figure S2(a)). KDQ performs in a manner similar to KDL except for some mild waves at the high end (Figure 4(b) and Additional file 2, Figure S2(b)). In contrast to the above three approaches, GRSN is implemented after the probe set summarization step with RMA, making it a second normalization. In our analysis, we replaced RMA with GCRMA due to its ability to improve the results of GRSN. GRSN improves the original GCRMA, as seen in Figure 4(c), although it does not resolve the heterogeneity between 1× genes and empty genes well. (Additional file 2, Figure S2(c)).

According to the above observations, the proposed KDL method captures the correct "invariant set" throughout the range of all intensity levels, outperforming the other methods. The major difference is the result of the bipartite pattern of null genes, comprising both 1× spike-in genes and empty genes. These two groups appear entirely different, making it impossible to merge them as one with any normalization methods, except for KDL and KDQ. Because the empty genes are highly artificial, they are unlikely to form a large set in real world data; however, the robustness of KDL and KDQ with respect to special groups of genes can greatly reduce the concern of slightly enhanced influential data points.

### 2.2 ROC curves

Because the expected fold changes for spiked-in transcripts are known, the tradeoff between false positive and false negative detection can be compared using ROC curves. This study compares some of the most popular combinations of preprocessing, probe set summarization, and detection methods to understand the advantages of adopting approaches based on invariant sets. GCRMA is used for background correction in most of the normalization methods except for dChip; therefore, we selected median polish for our summarization step. The normalization step can be applied prior to probe set summarization using KDL, KDQ, or global quantile normalization, which is the default of GCRMA. The Li-Wong model works with the invariant set in dChip as a whole. Because one of the methods we compare is GRSN, and it is implemented after probe set summarization, we also applied the proposed KDL after probe set summarization. The final step is to make inference on differentially expressed genes. We demonstrated this using both fold change and a t-test.

#### Normalization prior to expression summary

**Figure 5:**
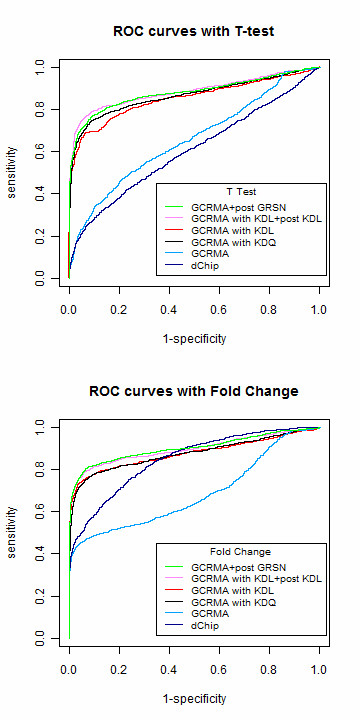
**ROC curves without the empty sets**. For the probe level data, quantile normalization is substituted with either KDL or KDQ in the workflow of GCRMA. They are shown as red and black curves, respectively. Original GCRMA is in light blue. The upper panel uses fold change and the lower panel uses T-test as the criterion to select differentially expressed genes. The two post summary normalizations are GRSN and double KDL, which adopts KDL normalization at both probe level and post summary level. They are shown as pink and green curves, respectively.

As shown in Figure 5 and Additional file 3, the ROC curve with either KDL or KDQ outperformed the original GCRMA, which adopted quantile normalization. The area under the ROC curve for dChip is smaller than the original GCRMA with t-test; however, this is the opposite for fold change. One issue related to Figure 5 concerns KDL and KDQ in the t-test panel. According to GCRMA version 2.16.0, probe intensities at very low levels are set to minimum values specific to each array in the background correction step, and the data become truncated below this value (Additional file 4). More than 40% of the data are truncated in the full data set of the Golden Spike experiment, and approximately 10% of the data if empty probes are not considered. If a probe remains at the low end across all six arrays, it will be assigned the same normalized intensity for all six arrays using loess normalization with smoothing parameters set at 0.2. Truncated probes are found mostly in empty sets; therefore, they have less influence on the data without empty genes. Such probe sets do not provide any variation for statistical testing because the summarized intensity for the six arrays is identical. This issue is inherited by KDL, which is based on loess, and this is also the case for quantile normalization. Hence, we set the p-values for such probe sets to 1 when drawing the ROC curves for the t-test.

#### Normalization after expression summary

When the Golden Spike experiment was first published [4], post summary normalization was recommended, for its ability to improve overall results. We investigated this option by performing a second normalization with both KDL and GRSN after GCRMA or dChip. The ROC curves with GCRMA are plotted in Figure 5 and Additional file 3. GRSN and KDL both improved GCRMA by a significant margin. We compared the ROC curves with respect to dChip in Additional file 5. Because dChip, GRSN, and KDL are all based on the idea of invariant sets, the second normalization does not improve dChip considerably.

The results in Figure 5 suggest that post-summary normalization with an invariant-set-based method such as KDL or GRSN applied after regular GCRMA does improve analysis. However, once an asymmetric pattern has been captured by the normalization method at the probe level, and adjusted in the right direction, there is no compelling need for a second normalization after the expression summary.

### 2.3 Expected fold change versus observed fold change

**Figure 6:**
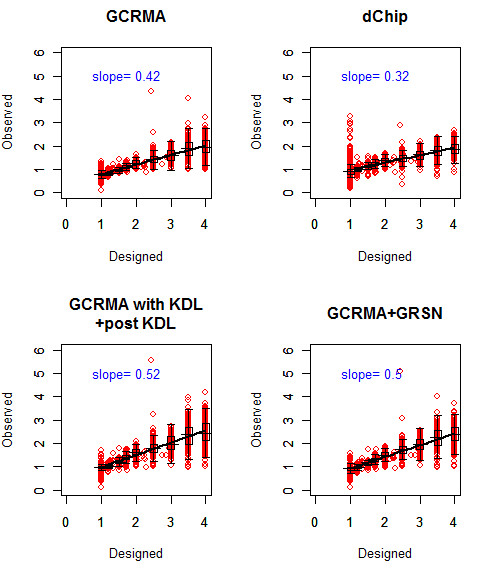
**Expected fold change versus the observed fold change for GCRMA, dChip, GCRMA with second normalization by KDL and GCRMA with second normalization by GRSN**.

ROC curves are used to compare the performance of different normalization methods in terms of both sensitivity and specificity from the perspective of ranking. Accurate estimation of true expression level is also desirable. Figure 6 compares designed fold change versus observed fold change following normalization. Regression lines are fitted for probe sets with designed fold change greater than one. Empty genes are excluded because too many influential points from the empty genes may bias the regression. We were primarily interested in the compression rate between the expected fold change and observed fold change, reflected by the slope of the regression line. The larger the slope is, the less the compression is. The dChip approach provides the highest compression rate among those compared. KDL normalization improved on GCRMA, with the slope of the regression line increasing from 0.42 to 0.52.

### 2.4 Simulation

We have demonstrated that KDL and KDQ are both able to improve the bias caused by asymmetry between the number of up- and down-regulated genes. However, it is important to determine at what cost this gain in performance is achieved, under conditions other than those specific to this scenario. Hence, we conducted a simulation study to discover whether the proposed methods could achieve the same level of performance with symmetrical expression change. We also evaluated the effects of altering parameters through simulation.

The simulation was based on the study by Gadbury et al.[22], in which microarray data was simulated from reference samples of a real data set. The idea was to borrow the real effect sizes from the full experiment with the base line constructed on null data. We followed this idea using a set of data downloaded from the GEO database http://www.ncbi.nlm.nih.gov/geo/ accession number GSE5788 [23], for the simulation. This experiment comprised six cases and eight controls run on the Affymetrix HG-U133A platform. The simulation steps were as follows.

1. Effect size distribution: We first summarized the 14 arrays with RMA [24] and then calculated the t-statistics for each of the 22283 probe sets. The distribution was centered on zero and demonstrated a high degree of symmetry.

2. We randomly separated the eight control samples into two groups of four samples each, assigning one group to be a simulated control group and the other the simulated treatment group.

3. We randomly selected a proportion of probe sets as simulated significant genes. We used 10%, 5%, and 1% of the total number of genes to generate results.

4. We randomly selected another set with the same number of probe sets and recorded the t-statistics derived in Step 1, as the simulated effect sizes.

5. For each probe set selected in Step 3, we calculated the standard deviation S_i _of the *i*th probe at the log scale across the simulated control samples. We then added the number S_i_xd_i _to the log of the *i*th probe of the simulated treatment samples, where d_i _was the *i*th simulated effect size selected in Step 4. We took this as the exponent of 2 to set the expression level back to the original scale.

All of the methods were then compared with ROC curves using the probe level data simulated above. The results are presented in Additional files 6, 7 and 8. There was essentially no difference between the methods with the well-behaved data. The best performance was achieved using post summary normalization strategies with GRSN and KDL.

**Figure 7:**
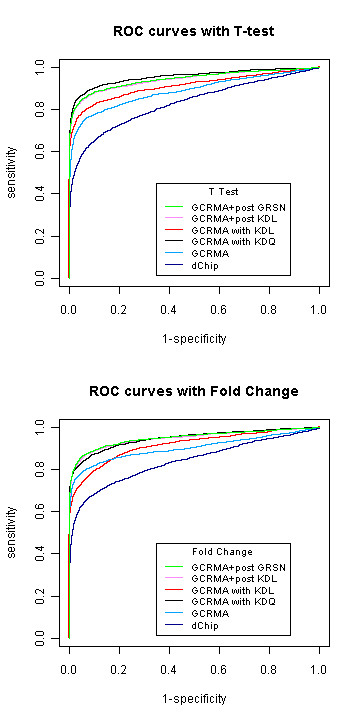
**ROC curves on simulation data with asymmetric expression change**. The data is simulated with 10% of significant genes as described in the context. All the treatment effects are positive.

Taking the absolute value of the effect sizes selected in Step 4 generates data with asymmetrical effects similar to those in the Golden Spike experiment. We compared all of the methods based on invariant sets with the ROC curves in Figure 7 using the asymmetrical simulation data. The proportion of significant genes was 10%, and the results related to different proportions can be found in Additional files 9 and 10. The previously mentioned properties still hold, in which the proposed KDL and KDQ improved GCRMA, regardless of whether they were applied before or after the probe set summary. The greater the asymmetric bias is, the larger the improvement that KDQ and KDL can achieve.

To better understand the influence of the tuning parameters on either method, we compared the results with the same simulation data using a variety of settings. The data was simulated with either 1% or 10% significant genes, and all treatment effects were positive. We first assessed the power of the kernel density used in the weighting scheme of KDL. Additional files 11 and 12 present the ROC curves with different multiplicity settings in KDL when integrated with GCRMA. Very little difference was observed for multiplicity ranging from one to five. The impact of the settings increased with the proportion of significant genes, and greater multiplicity was preferred under these conditions. For post summary normalization with KDL, multiplicity has nearly no influence on detection ability. Additional files 13 and 14 present the ROC curves for KDL as a post summary normalization with GCRMA. The second parameter we assessed was the proportion of invariant sets in KDQ. We tried various proportions from 40% to 90%. Additional files 15 and16 show that, with a higher degree of asymmetry in the data, we should select a conservative proportion of invariant sets to ensure that deregulated genes are not included in the invariant set.

## 3. Discussion

The design of the Golden Spike experiment provided a good opportunity to review existing normalization methods and determine how they handle asymmetric changes in expression levels. Although it is an extreme design, the information provided is exceptional, compared to other spike-in studies.

This study demonstrated the superiority of methods based on invariant sets, compared to global normalization methods for data with an asymmetric expression structure. The two proposed normalization strategies based on kernel density, KDL and KDQ, improve the popular normalization methods, loess and quantile normalization, at either the probe-level or post-summary level. Both strategies can be integrated with any procedures containing an independent normalization step.

As with most normalization strategies, a number of parameters in the proposed method require a degree of tuning. As with loess normalization, the smoothing parameter was set to 0.2 in this study. Local structure can be captured using smaller values. In addition, we used density estimates to the power of four as weights in the loess normalization for KDL, although this could certainly be set higher or lower to put more or less emphasis on the data in the middle of the major trend. This multiplicity plays an important role when asymmetry is strong.

KDL is applied to the entire data set with a weighting scheme while KDQ adopts an approach similar to that of other invariant-set normalization methods. This is accomplished by initially selecting a subset of data to be normalized and extending the normalization process to the other data not included in the selected subset. The selection of subset is based on kernel density estimation as well as knowledge related to the proportion of genes expected to be differentially expressed across arrays. Selecting the proportion of genes in the training subset should be based on a thorough understanding of the data. According to our empirical study and simulation, a conservative proportion would prevent the inclusion of any variable genes, particularly in situation involving a high degree of asymmetry. In other respects, KDQ is quite robust with regard to this parameter.

In the above comparison with the Golden Spike experiment and simulation data, we found that KDQ performed slightly better than KDL in probe level normalization, although both of these methods produced similar results. Nevertheless, the soft weighting scheme of KDL automatically included all of the data points in the analysis, while KDQ relied on extrapolation to normalize data points outside the range of the invariant set. Hence, if special patterns are observed in the intensity-based curvature across arrays at the two ends of the data, it is preferable to adopt KDL over KDQ.

The steps of preprocessing and expression summary play an important role in determining the accuracy of estimation. The statistics for the detection of differentially expressed genes further improves the retrieval of correct signals from noise. This study does not compare all possible combinations of analysis strategies to provide the best suggestion, as this would be impractical. Rather, we aimed to demonstrate the importance of dealing with asymmetric patterns in the normalization step and how methods based on invariant sets are better able to improve existing methods. If the characteristics of the data are accurately detected and dealt with, statistical testing for inference will proceed far more smoothly. The proposed KDL and KDQ methods are presented as improved substitutes for both loess and quantile normalization in general, and more specifically for integration with GCRMA.

## 4. Conclusion

The proposed KDL approach is a simple strategy to improve the accuracy of GCRMA in estimation. Based on our results using t-test and fold change, it is clear that detection power is enhanced; therefore, it is highly recommended for the routine practice of microarray data analysis. Both KDL and KDQ are implemented in JMP Genomics Version 5.0.

## 5. Methods

### 5.1 Golden Spike Experiment

To demonstrate the strength of the proposed methods, we used a dataset with spiked-in transcripts of known concentrations. The Golden Spike experiment [4] has been discussed and used to compare methodologies in many studies [25-27]http://www2.ccr.buffalo.edu/halfon/spike/spikedownloads.html. The Golden Spike experiment was the first, and one of the few entirely controlled experiments with known transcript levels for every gene in the array. The experiment consists of three replicates for the S group and three replicates for the C group using DrosGenome1 GeneChip. The S group includes 1331 spiked-in transcripts with higher concentrations than C group, and 2535 spiked-in transcripts of equal concentration in the two groups. The other 10144 probe sets in the arrays are empty sets that do not target any spiked-in sequences.

Criticism of this data generally relates to the confounding effects between transcript level and fold change, (assigning larger fold changes to higher intensity probes), an unfair experimental design, asymmetry in gene effects toward up-regulation and the existence of an unusually large empty set [16,18,21]. However, confounding effects also exist in real data. Researchers have often observed in MA plots that a gene expressed at a higher level has a strong effect when differentially regulated. It is unclear whether this stronger effect is caused by artifacts from the normalization step or represents an actual biological phenomenon. Issues related to design can be found in nearly every microarray experiment. This issue is not limited to spike-in experiments and most two group comparisons suffer from such problems because experiments cannot be perfectly controlled. Such systemic variations must be corrected before any inference can be made about differential expression. Furthermore, this data set is particularly well suited to assessing sensitivity to violations of symmetric assumptions.

### 5.2 Kernel Density weighted Loess normalization (KDL)

The kernel method is a non-parametric technique for the estimation of density. Let *f (x, y) *be the joint density function of the bivariate random variable (*X*, *Y*), and let (*X_i _*, *Y_i _*), i = 1, ⋯, *n *be a sample of size *n *drawn from this distribution. The kernel density estimate of *f (x, y) *based on this sample is

where *h_X _*> 0 and *h_Y _*> 0 are the bandwidths and *ϕ(x,y) *is the standard normal density.

Ni et al. proposed a kernel density based method, fitting kernel density to the M values restricted to points within a particular range of A values, where M stands for the difference between the two compared samples and A stands for the mean intensity of the two samples. The mode of one-dimensional kernel estimation is used to represent the bias of effect size on non-differentially expressed genes within each region of A, and the normalization curve is derived by connecting the modes across various regions of A with smoothing splines. This two-step approach requires a number of empirical parameters with which to select modes.

Unlike the method of Ni et al., our approach measures density in two dimensions (M and A) jointly, and loess normalization is used to fit polynomial curves locally with the data. Points proximal to one another along the x axis jointly determine the main trend of the curve. KDL simply assigns different weights to the data points according to the estimated kernel density when fitting the loess curve. This paper applied the estimated kernel density to the power of 4, as the weight of the corresponding probe. Genes deviating from the major group, which consists of null genes, are down-weighted. The weighting strategy of KDL benefits from not having to establish any hard decisions in the selection of the invariant set, while allowing the normalization process to rely heavily on probe intensities that remain consistent across arrays. Because all of the probes are included throughout the normalization process, the common issue of having to extrapolate scores for methods based on invariant sets does not exist in KDL.

The only requirement of KDL is that null genes be distributed more closely than others. This is true in most empirical studies, but the Golden Spike experiment violates this assumption by including two distinct null sets, 1× genes and empty genes. These genes do not perform like a single group, and therefore distort most of the normalization strategies. We will discuss the results of both including and excluding empty genes.

The SAS procedures, PROC KDE and PROC LOESS (SAS Institute Inc., 2009), are used for estimating kernel density and loess model fitting. First, the average of all arrays is taken as a common reference. Each array is normalized against the reference by fitting the weighted loess curve to the MA plot. The A dimension is employed as reference data, rather than using the mean between the target array and reference array. This slight difference provides an enormous advantage in resolving heterogeneity in data, as shown in Section 2.1.

### 5.3 Kernel Density Quantile normalization (KDQ)

Kernel density estimation can also be used to select invariant sets, which should include the null genes. We integrated this idea with quantile normalization. The global normalization strategy associated with quantile normalization can be adjusted for asymmetric data. The proposed steps are as follows.

#### Step 1. Invariant set selection

We first apply kernel density estimation to the MA plot between each array and the reference array. The reference array can be objectively selected or set to the average of all of the arrays. As with KDL, we assign the common average among all arrays as the A component. The M component represents the difference between each individual array and the reference array. The estimation of density is a score showing the relative importance of each data point in the plot. Probes with greater importance scores are included in the invariant set. Similar to other invariant set selection methods, there are always a number of thresholds that must be determined. KDQ requires prior knowledge with which to establish the proportion of data included in the invariant set. It is recommended that this proportion be set lower than but close to the expected proportion of probes without differential expression across arrays. For the Golden Spike experiment, we set this proportion to 85% and 50% including empty null genes and excluding empty null genes, respectively. This tuning parameter is sensitive and data dependent.

#### Step 2. Quantile normalization with the invariant set

The second step involves conducting the general quantile normalization procedure on the invariant set for all of the arrays.

#### Step 3. Scoring the non-invariant set

Data not included in the invariant set has to be scored relative to the invariant set. For data within the range of the invariant set, linear interpolation is applied within each array. For data outside the range of the invariant set, linear extrapolation is adopted, based on a small set of data at the boundary of the invariant set. To clarify this point, we will illustrate it with an example. Assume that the intensities of *n *probes in the invariant set are *x*_1_, *x*_2_, ..., *x*_n_. Their order statistics are *x*_(1)_, *x*_(2)_, ..., *x*_(n)_. The corresponding normalized intensities from Step 2 are y_(1)_, *y*_(2)_, ..., *y*_(n) _ordered from smallest to largest. When considering a probe that is not in the invariant set with intensity *x *falling between *x*_(i) _and *x*_(i+1)_, the interpolated value will be *y*_(i)_+(*x-x*_(i)_)x(*y*_(i+1)_*-y*_(i)_)/(*x*_(i+1)_*-x*_(i)_). If *x *is greater than *x*_(n)_, extrapolation will depend on the highest *m *values of the invariant set. In this case, *m *is set to 100. Let *x*_a _be the average of *x*_(n-99)_, *x*_(n-98)_, ..., *x*_(n) _and *y*_a _be the average of *y*_(n-99)_, *y*_(n-98)_, ..., *y*_(n)_. The normalized value for *x *will be *y*_a_*+*(*x-x*_a_). This is only adjusted for the average shift between the original and the normalized data of the rightmost 100 observations in the invariant set.

#### 5.4 Probe level normalization and post summary normalization

Our proposed normalization based on kernel density can be applied to probe level data as well as probe set level data, and these have been summarized for each probe set in Affymetrix GeneChip data. The implementation of post summary normalization is straightforward, as described in Sections 5.2 and 5.3. For probe level data, a small twist is made. We first take the median of each probe set and apply normalization to the medians. The probe level data is then reconstructed by adding back the corresponding difference of each probe relative to the median.

Both KDL and KDQ were implemented in SAS 9.2 (SAS Institute, Cary, NC) and integrated into JMP Genomics Version 5.0 (SAS Institute, Cary, NC). The SAS codes and corresponding SAS dataset are available at: http://www.stat.nthu.edu.tw/~wphsieh/KD.htm.

## Authors' contributions

WPH, TMC and RDW conceived the project. TMC implemented KDL and KDQ. WPH and YML did the comparison and simulation. WPH, TMC, YML and RDW drafted the manuscript. All authors read and approved the final manuscript.

## Supplementary Material

Additional file 1 **Figure S1 - MA plots for probe-level normalization using data with the empty genes**. (a) Data are normalized with quantile normalization and summarized with the median of each probe set. (b) Data are normalized with loess normalization and summarized with the median of each probe set. (c) Data are first background-corrected with GCRMA and normalized with quantile normalization. The expression summary is then computed using median polish. (d) Data are first background-corrected with GCRMA and normalized with loess normalization. The expression summary is then computed using median polish. The blue points are empty genes, the red points are the 1× genes and the green ones are spiked with higher concentration in the S group than in the C group. Click here for file

Additional file 2 **Figure S2 - MA plots for invariant set based normalization methods using data with the empty genes**. (a) Data are background-corrected with GCRMA and normalized at the probe level with KDL. Median polish is used to summarize the probe set expression level. (b) The same as (a) while replacing the normalization with KDQ. (c) Data are background-corrected with GCRMA and normalized at the probe level with quantile. Median polish is used to summarize the probe set expression level. The data are then normalized again at the post summary level with GRSN. (d) Li and Wong's dChip method implemented in R. Click here for file

Additional file 3 **Figure S3 - ROC curves with the empty sets**. For the probe level data, quantile normalization is substituted with either KDL or KDQ in the workflow of GCRMA. They are presented in red and black respectively. The original GCRMA is in light blue. The upper panel uses T-test and the lower panel uses Fold Change as the criterion to select differentially expressed genes. They both report similar orders of performance. The two post summary normalizations, KDL and GRSN, are presented in pink and green respectively. Click here for file

Additional file 4 **Figure S4 - Scatter plots for the probe data after GCRMA background correction**. The vertical and horizontal lines at left bottom coner of each plot indicate certain truncation applied for the low-end intensities. Click here for file

Additional file 5 **Figure S5 - ROC curves related to dChip with empty genes**. The ROC curve derived from dChip is in blue. GRSN and KDL are then applied on the dChip data as the second normalization and are shown as green and red curves, respectively. The upper panel uses T-test and the lower panel uses Fold Change as the criterion to select differentially expressed genes. Click here for file

Additional file 6 **Figure S6 - ROC curves on simulation data with symmetric expression change**. The data was simulated with 1% of significant genes as described in the context. The treatment effects could be either positive or negative. The curves for GCRMA, GCRMA + post GRSN and GCRMA + post KDL are closely overlapped. Click here for file

Additional file 7 **Figure S7 - ROC curves on simulation data with symmetric expression change**. The data was simulated with 5% of significant genes as described in the context. The treatment effects could be either positive or negative. The curves for GCRMA, GCRMA + post GRSN and GCRMA + post KDL are closely overlapped. Click here for file

Additional file 8 **Figure S8 - ROC curves on simulation data with symmetric expression change**. The data was simulated with 10% of significant genes as described in the context. The treatment effects could be either positive or negative. The curves for GCRMA, GCRMA + post GRSN and GCRMA + post KDL are closely overlapped. Click here for file

Additional file 9 **Figure S9 - ROC curves on simulation data with asymmetric expression change**. The data was simulated with 1% of significant genes as described in the context. All the treatment effects were positive. Click here for file

Additional file 10 **Figure S10 - ROC curves on simulation data with asymmetric expression change **. The data was simulated with 5% of significant genes as described in the context. All the treatment effects were positive. Click here for file

Additional file 11 **Figure S11 - GCRMA with KDL on asymmetrical data generated with 1% of significant genes**. The data was simulated with 1% of significant genes as described in the context. All the treatment effects were positive. Click here for file

Additional file 12 **Figure S12 - GCRMA with KDL on asymmetrical data generated with 10% of significant genes**. The data was simulated with 10% of significant genes as described in the context. All the treatment effects were positive. Click here for file

Additional file 13 **Figure S13 - GCRMA + post KDL on asymmetrical data generated with 1% of significant genes**. The data was simulated with 1% of significant genes as described in the context. All the treatment effects were positive. Click here for file

Additional file 14 **Figure S14 - GCRMA + post KDL on asymmetrical data generated with 10% of significant genes**. The data were simulated with 10% of significant genes as described in the context. All the treatment effects were positive. Click here for file

Additional file 15 **Figure S15 - GCRMA with KDQ on asymmetrical data generated with 1% of significant genes**. The data was simulated with 1% of significant genes as described in the context. All the treatment effects were positive. Click here for file

Additional file 16 **Figure S16 - GCRMA with KDQ on asymmetrical data generated with 10% of significant genes**. The data was simulated with 10% of significant genes as described in the context. All the treatment effects were positive. Click here for file
