# Supplementary figures and images for: Kernel density weighted loess normalization improves the performance of detection within asymmetrical data
Source: BMC Bioinformatics. 2011 Jun 1;12:222. doi: 10.1186/1471-2105-12-222 (PMC3118355; doi:10.1186/1471-2105-12-222)

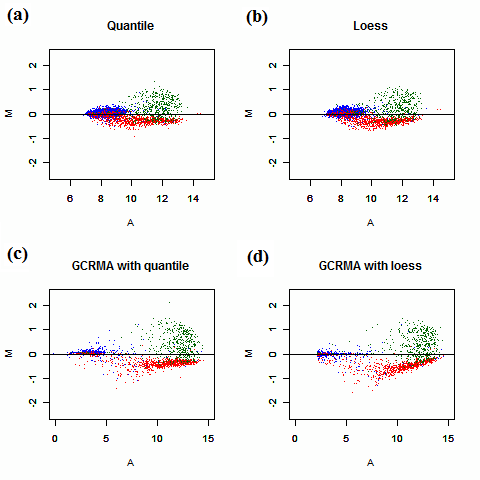

Supplement: Additional file 1 — Figure S1 - MA plots for probe-level normalization using data with the empty genes. (a) Data are normalized with quantile normalization and summarized with the median of each probe set. (b) Data are normalized with loess normalization and summarized with the median of each probe set. (c) Data are first background-corrected with GCRMA and normalized with quantile normalization. The expression summary is then computed using median polish. (d) Data are first background-corrected with GCRMA and normalized with loess normalization. The expression summary is then computed using median polish. The blue points are empty genes, the red points are the 1× genes and the green ones are spiked with higher concentration in the S group than in the C group. [file 1471-2105-12-222-S1.PNG]

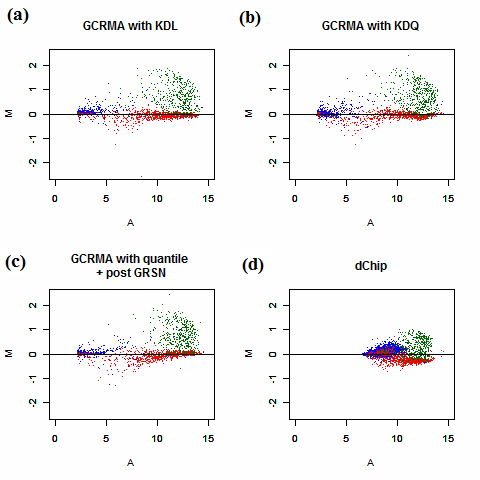

Supplement: Additional file 2 — Figure S2 - MA plots for invariant set based normalization methods using data with the empty genes. (a) Data are background-corrected with GCRMA and normalized at the probe level with KDL. Median polish is used to summarize the probe set expression level. (b) The same as (a) while replacing the normalization with KDQ. (c) Data are background-corrected with GCRMA and normalized at the probe level with quantile. Median polish is used to summarize the probe set expression level. The data are then normalized again at the post summary level with GRSN. (d) Li and Wong's dChip method implemented in R. [file 1471-2105-12-222-S2.PNG]

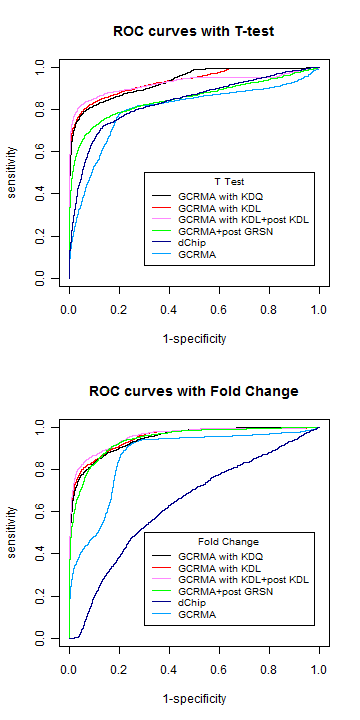

Supplement: Additional file 3 — Figure S3 - ROC curves with the empty sets. For the probe level data, quantile normalization is substituted with either KDL or KDQ in the workflow of GCRMA. They are presented in red and black respectively. The original GCRMA is in light blue. The upper panel uses T-test and the lower panel uses Fold Change as the criterion to select differentially expressed genes. They both report similar orders of performance. The two post summary normalizations, KDL and GRSN, are presented in pink and green respectively. [file 1471-2105-12-222-S3.PNG]

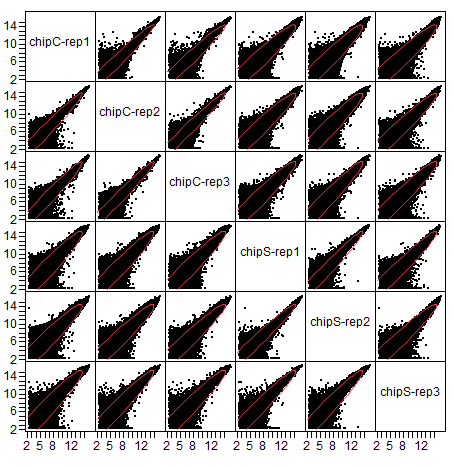

Supplement: Additional file 4 — Figure S4 - Scatter plots for the probe data after GCRMA background correction. The vertical and horizontal lines at left bottom coner of each plot indicate certain truncation applied for the low-end intensities. [file 1471-2105-12-222-S4.PNG]

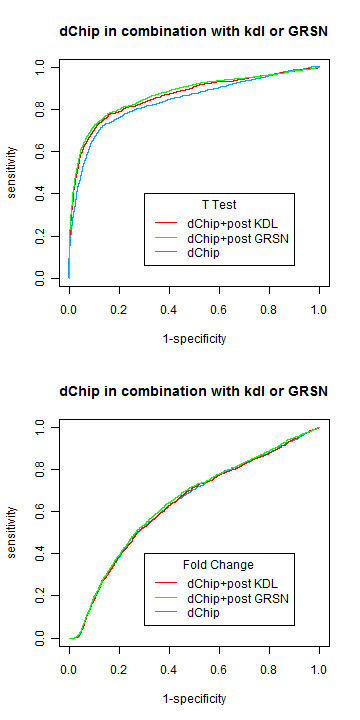

Supplement: Additional file 5 — Figure S5 - ROC curves related to dChip with empty genes. The ROC curve derived from dChip is in blue. GRSN and KDL are then applied on the dChip data as the second normalization and are shown as green and red curves, respectively. The upper panel uses T-test and the lower panel uses Fold Change as the criterion to select differentially expressed genes. [file 1471-2105-12-222-S5.PNG]

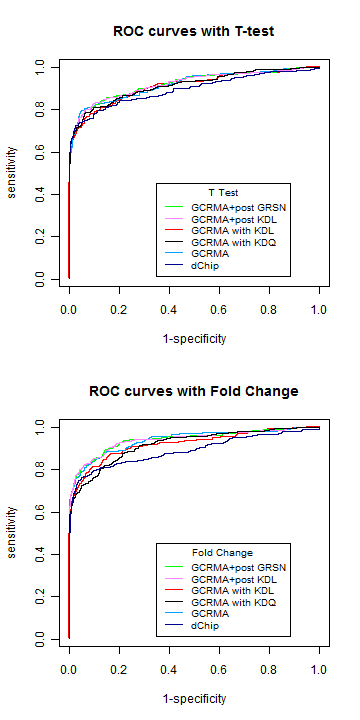

Supplement: Additional file 6 — Figure S6 - ROC curves on simulation data with symmetric expression change. The data was simulated with 1% of significant genes as described in the context. The treatment effects could be either positive or negative. The curves for GCRMA, GCRMA + post GRSN and GCRMA + post KDL are closely overlapped. [file 1471-2105-12-222-S6.PNG]

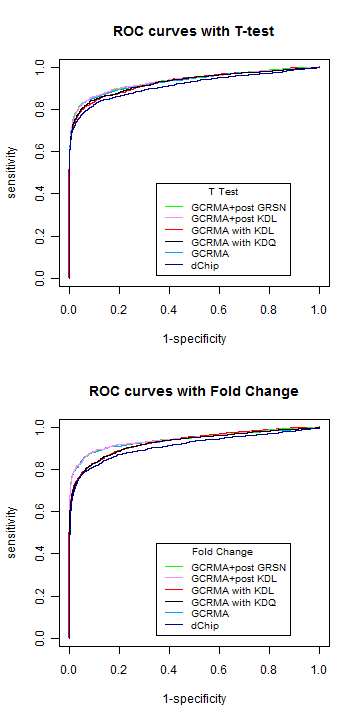

Supplement: Additional file 7 — Figure S7 - ROC curves on simulation data with symmetric expression change. The data was simulated with 5% of significant genes as described in the context. The treatment effects could be either positive or negative. The curves for GCRMA, GCRMA + post GRSN and GCRMA + post KDL are closely overlapped. [file 1471-2105-12-222-S7.PNG]

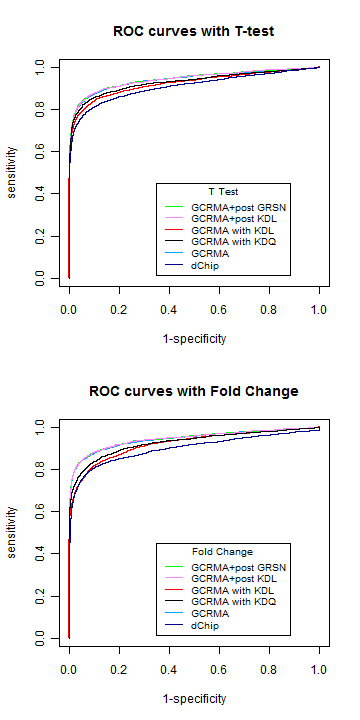

Supplement: Additional file 8 — Figure S8 - ROC curves on simulation data with symmetric expression change. The data was simulated with 10% of significant genes as described in the context. The treatment effects could be either positive or negative. The curves for GCRMA, GCRMA + post GRSN and GCRMA + post KDL are closely overlapped. [file 1471-2105-12-222-S8.PNG]

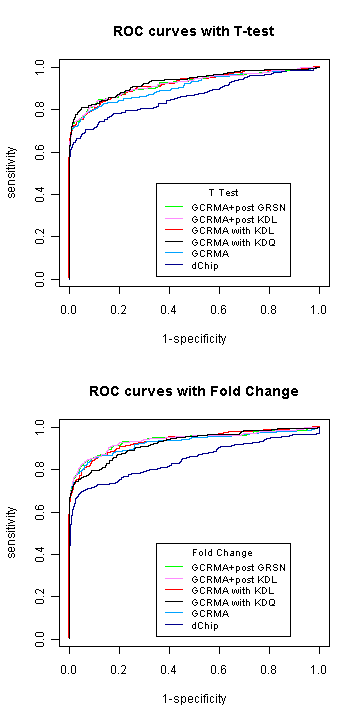

Supplement: Additional file 9 — Figure S9 - ROC curves on simulation data with asymmetric expression change. The data was simulated with 1% of significant genes as described in the context. All the treatment effects were positive. [file 1471-2105-12-222-S9.PNG]

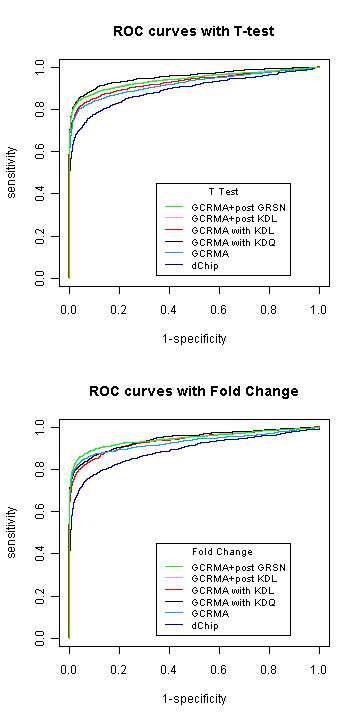

Supplement: Additional file 10 — Figure S10 - ROC curves on simulation data with asymmetric expression change . The data was simulated with 5% of significant genes as described in the context. All the treatment effects were positive. [file 1471-2105-12-222-S10.PNG]

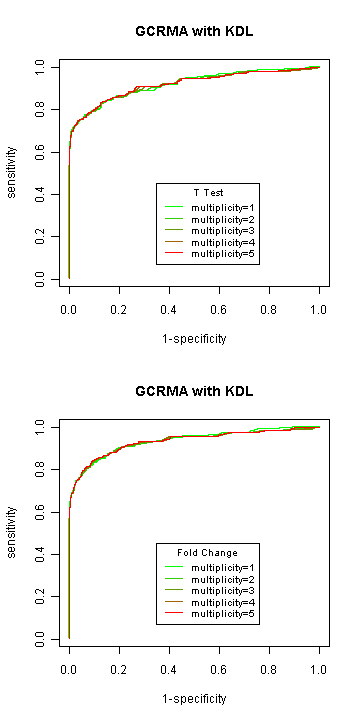

Supplement: Additional file 11 — Figure S11 - GCRMA with KDL on asymmetrical data generated with 1% of significant genes. The data was simulated with 1% of significant genes as described in the context. All the treatment effects were positive. [file 1471-2105-12-222-S11.PNG]

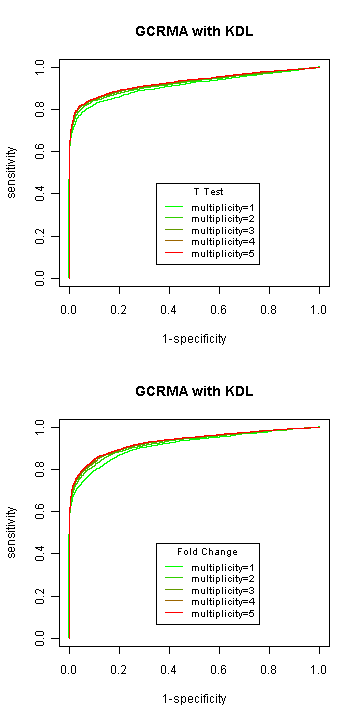

Supplement: Additional file 12 — Figure S12 - GCRMA with KDL on asymmetrical data generated with 10% of significant genes. The data was simulated with 10% of significant genes as described in the context. All the treatment effects were positive. [file 1471-2105-12-222-S12.PNG]

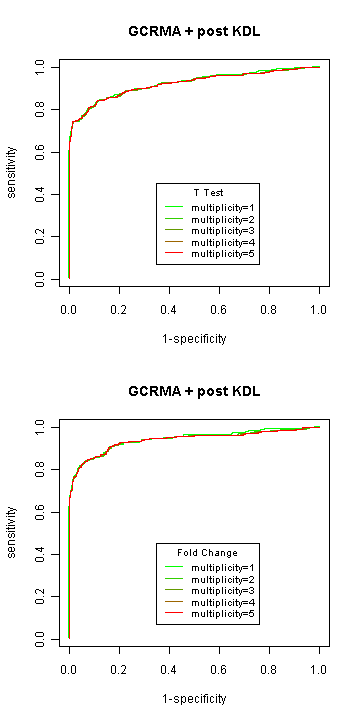

Supplement: Additional file 13 — Figure S13 - GCRMA + post KDL on asymmetrical data generated with 1% of significant genes. The data was simulated with 1% of significant genes as described in the context. All the treatment effects were positive. [file 1471-2105-12-222-S13.PNG]

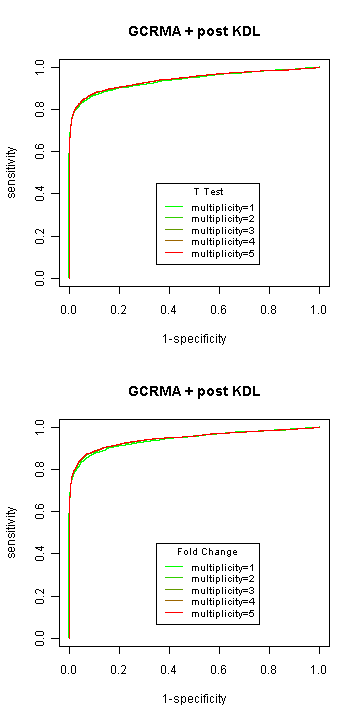

Supplement: Additional file 14 — Figure S14 - GCRMA + post KDL on asymmetrical data generated with 10% of significant genes. The data were simulated with 10% of significant genes as described in the context. All the treatment effects were positive. [file 1471-2105-12-222-S14.PNG]

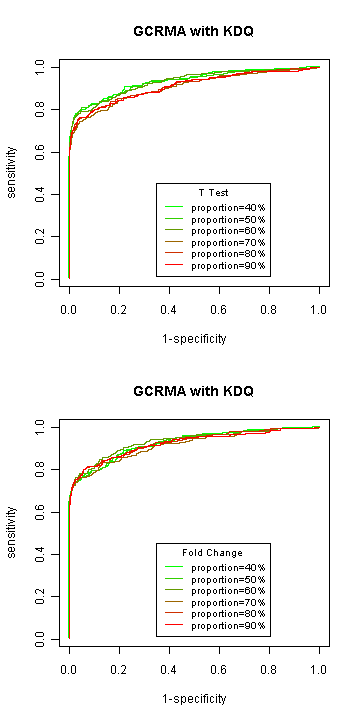

Supplement: Additional file 15 — Figure S15 - GCRMA with KDQ on asymmetrical data generated with 1% of significant genes. The data was simulated with 1% of significant genes as described in the context. All the treatment effects were positive. [file 1471-2105-12-222-S15.PNG]

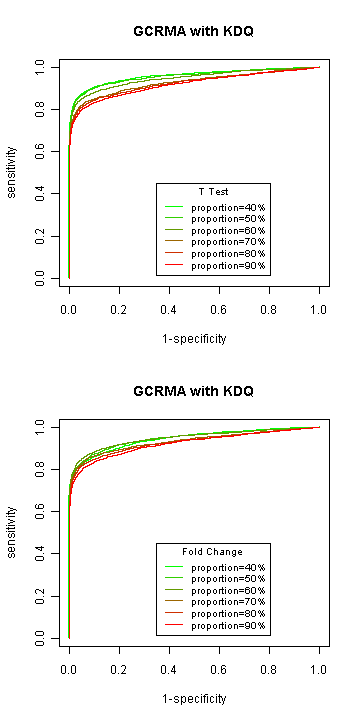

Supplement: Additional file 16 — Figure S16 - GCRMA with KDQ on asymmetrical data generated with 10% of significant genes. The data was simulated with 10% of significant genes as described in the context. All the treatment effects were positive. [file 1471-2105-12-222-S16.PNG]
